# Auxetic Composite Laminates with Through-Thickness Negative Poisson’s Ratio for Mitigating Low Velocity Impact Damage: A Numerical Study

**DOI:** 10.3390/ma15196963

**Published:** 2022-10-07

**Authors:** Yeqing Wang

**Affiliations:** Department of Mechanical & Aerospace Engineering, Syracuse University, Syracuse, NY 13244, USA; ywang261@syr.edu; Tel.: +1-(315)-443-2341

**Keywords:** auxetic composite laminates, carbon fiber composite, negative Poisson’s ratio, low velocity impact, finite element analysis

## Abstract

Auxetic materials are those that exhibit negative Poisson’s ratios. Such a unique property was shown to improve the indentation and impact resistances. Angle-ply composite laminates can be designed to produce negative Poisson’s ratio at the laminate level due to the large anisotropicity of the individual layer and the strain mismatch between adjacent layers. This paper investigates the effect of through-thickness negative Poisson’s ratio on the low velocity impact behaviors of carbon fiber reinforced polymer matrix composite laminates, including the global impact behaviors, as well as the delamination, and the fiber and matrix damage. Results from numerical investigations show consistently reduced fiber and matrix tensile damage in the auxetic laminate in all plies, in comparison to the non-auxetic counterpart laminates (up to 40% on average). However, the auxetic laminate does not present a clear advantage on mitigating the delamination damage or the matrix compressive damage.

## 1. Introduction

Carbon fiber reinforced polymer (CFRP) matrix composites are increasingly used in a variety of industries, such as aerospace, marine, automotive, energy, civil infrastructure, and high-end sports. This is due to their significant weight-saving capability and extraordinary properties, including the high specific stiffness and specific strength, excellent fatigue and corrosion resistance, and low coefficient of thermal expansion. However, despite having extraordinary properties, these composites are susceptible to the low velocity impact of foreign objects in service life (e.g., tool drop impact, and the impact of debris from the runway) [1,2,3,4,5]. The impact will cause delamination, matrix cracking, and fiber breakage, which results in significant degradations in mechanical properties [6,7,8,9]. For example, the compression strength after impact experienced a reduction of 34.5% and 60.2% for a 16-layer CFRP composite plate, when subjected to an impact energy of 17 J and 29.5 J, respectively [10].

One potential approach to improve the low velocity impact damage tolerance of CFRP composites is to exploit the laminate-level negative Poisson’s ratios. Materials or structures that exhibit negative Poisson’s ratios are known as auxetic materials or structures [11]. Typically, materials contract transversely under uniaxial tension and expand transversely under uniaxial compression. Auxetic materials/structures exhibit counterintuitive behavior as they expand transversely after tension and contract transversely after compression. Such materials/structures are rare in nature, and thus, are often artificially engineered. There are several ways of engineering auxetic structures. The most common way is to use porous structures, such as re-entrant or chiral structures [12,13,14]. Another way is to use non-porous multidirectional layered composite structures [15,16,17,18]. Additionally, auxetic materials and structures have been developed and studied on different levels and various length scales [19,20,21,22,23,24,25,26,27,28,29,30,31,32]. Examples include auxetic structures that are achieved at the molecular level [23] and through microscopic structure modification [24]. Moreover, researchers have also studied cubic metals that exhibit auxetic behaviors [27] and methods to tailor graphene to achieve negative Poisson’s ratio [31]. The current study focuses on the non-porous multidirectional layered composite structures, which are designed to produce negative Poisson’s ratio at the macroscopic continuum level.

**Figure 1:**
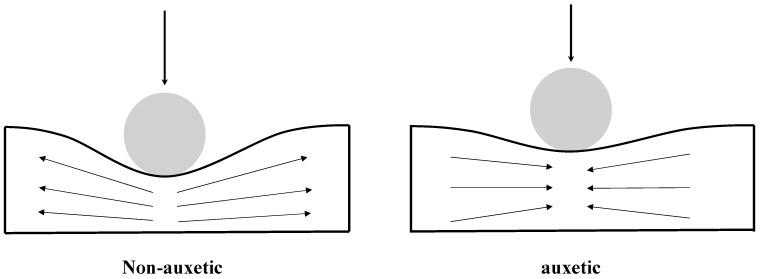
A comparison of indentation behavior between a non-auxetic material (**left**) and an auxetic material (**right**) [41].

Previous studies have shown that auxetic materials/structures demonstrate performance enhancements in many properties, such as indentation resistance [13,33], impact resistance [13,15,16,34,35,36], energy absorption [37], shock wave absorption [38], and the sensitivity of strain sensing [39,40], when compared to their non-auxetic counterparts. For example, the experimental test data reported by Ref. [13] showed that the indentation stiffness of an auxetic lattice reinforced composite structure with a Poisson’s ratio of −0.4 is three times that of the composite with a positive Poisson’s ratio of 1. Figure 1 shows a comparison of the indentation behaviors between non-auxetic and auxetic materials. Specifically, the through-thickness compression that occurs during mechanical impact will cause auxetic materials to contract in their in-plane directions. Material, therefore, flows into the vicinity of the impact site and the density locally increases. The result is that an auxetic material will have an improved capability to withstand the localized deformation, in comparison to an otherwise identical non-auxetic one.

Although experimental evidence exists, the number of corresponding modeling studies is scarce, especially for those focusing on the non-porous layered auxetic composite structures. The effect of laminate-level negative Poisson’s ratio on the global impact response and damage behavior of the composite structures remains largely unknown. Without such understanding, it is challenging to exploit the negative Poisson’s ratio as a design constraint to achieve controllable performance enhancements in layered composite structures. To fill this knowledge gap, numerical simulations are performed in this study to understand the effect of laminate-level negative Poisson’s ratio on the low velocity impact behavior of CFRP composite laminates at elevated impact energies. 

## 2. Layups of CFRP Composite Laminates That Allow to Produce Negative Poisson’s Ratios

By leveraging the anisotropicity of the individual CFRP composite ply (i.e., a large ratio between *E*_11_ and *E*_22_) and the strain mismatch between adjacent plies, laminate-level (or effective) negative Poisson’s ratios can be produced by tuning the layup of the composite laminates [18]. According to the Classical Lamination Theory, the laminate-level through-thickness Poisson’s ratio is [35,36],

(1) $$\nu_{13}^{e}=-\frac{J_{31}}{J_{11}}$$

where *J*_11_ and *J*_31_ are elements of the **J** matrix,

(2) $$J=A^{-1}+A^{-1}B{\left(D-BA^{-1}B\right)}^{-1}BA^{-1}$$

where **A**, **B**, and **D** are the extensional stiffness, extensional-bending coupling stiffness, and bending stiffness matrices [42,43]. 

**Figure 2:**
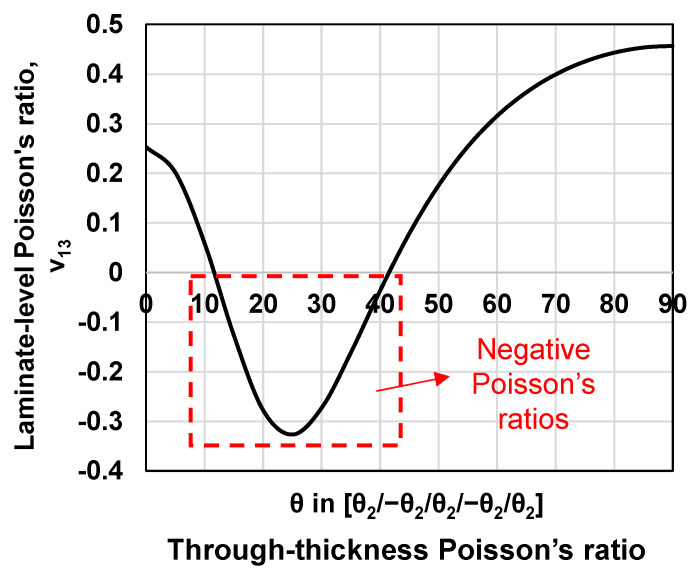
Predicted layups to produce laminate-level through-thickness negative Poisson’s ratios in IM7/977-3 CFRP composite laminates with a layup of [*θ*_2_/−*θ*_2_/*θ*_2_/−*θ*_2_/*θ*_2_] as the ply angle, *θ*, changes from 0 to 90 degrees.

As shown in Equations (1) and (2), the negative Poisson’s ratio reflects the combined effect of the **A**, **B**, and **D** matrices. Using these equations, the layups for laminated composites to produce negative Poisson’s ratios in the through-thickness direction can be identified. Note that fundamental coupon-level tensile tests, conducted by the author’s group [44] and other researchers [17,18,45,46], have proved that the laminate-level negative Poisson’s ratios predicted analytically, based on the **A**, **B**, and **D** matrices, agree favorably with the experimental data. Table 1 below shows the engineering constants of the IM7/977-3 CFRP composite laminate [47,48,49] that were used in the calculation of the **A**, **B**, and **D** matrices. Figure 2 shows the calculated through-thickness Poisson’s ratio, $\nu_{13}^{e}$, for the laminate with a layup of [*θ*_2_/−*θ*_2_/*θ*_2_/−*θ*_2_/*θ*_2_] as the ply angle, *θ*, changes between 0 and 90 degrees. As we can see, the negative through-thickness Poisson’s ratios are produced when the ply angle is between 15 and 40 degrees. The largest through-thickness negative Poisson’s ratio is found at the ply angle of 25 degrees. It is worth noting that the CFRP composite laminate is anisotropic. The layups marked in the red dashed box in Figure 2 only allow negative Poisson’s ratios in the through-thickness direction. In the in-plane directions, the Poisson’s ratios remain positive. Since the auxeticity is produced in only one direction, when adopting the terminology proposed by Branka et al. [50], these composite structures can be considered partially auxetic.

## 3. Layups of Non-Auxetic CFRP Counterpart Laminates with Positive Poisson’s Ratios

To ensure a meaningful comparison, the layups of the counterpart CFRP laminates are identified such that they allow them to produce positive Poisson’s ratios and, at the same time, produce identical effective moduli to those of the auxetic laminates. The effective moduli of the laminate are calculated using the well-validated analytical equations proposed by Sun and Li [42]. It is worth noting that no layups exist that would allow the non-auxetic counterpart laminate to 100% match all effective moduli in three directions of the auxetic composite laminate (i.e., $E_{1}^{eff}$, $E_{2}^{eff}$, and $E_{3}^{eff}$). Therefore, two matching configurations are used for best approximations: Configuration 1 strictly matches both the longitudinal and the through-thickness effective moduli ($E_{1}^{eff}$ and $E_{3}^{eff}$) of those of the auxetic laminate with very low tolerances (≤0.7%), and without constraining the transverse effective modulus ($E_{2}^{eff}$), while Configuration 2 is the best available configuration that matches all effective moduli of the auxetic laminate in three directions with relatively higher tolerances. Table 2 shows our identified layups of the non-auxetic counterpart composite laminates in both. The reason for choosing the layups of [25_2_/−25_2_/25_2_/−25_2_/25_2_] for the auxetic composite is that this layup will allow the laminate to produce the largest through-thickness negative Poisson’s ratio, as shown in Figure 2, which is expected to provide the most significant enhancement in the low velocity impact resistance. Note that, this layup is an unbalanced layup and does not necessarily represent an optimum layup for practical engineering applications. It is only used here to study the effect of the laminate-level negative Poisson’s ratio on the impact resistance of CFRP composites.

## 4. Low Velocity Impact Model for CFRP Composite Laminates

To study the effect of the laminate-level negative Poisson’s ratio on the low velocity impact behavior of the composite laminates, a well-validated progressive damage modeling approach [7,10,47,49,51,52,53] is used. The primary components of the low velocity impact model adopted throughout this study include: (1) the Hashin damage criteria, which are used to predict the initiation of the fiber tensile and compressive failure, and the matrix tensile and compressive failure [54]; (2) the linear stiffness degradation function, based on the equivalent strain method [49], which is used to track the damage evolution in each failure mode [49]; and (3) the Benzeggagh and Kenane (B—K) delamination criterion along with mixed-mode fracture energy laws, which are used to model the initiation and evolution of the delamination damage [55]. The low velocity impact model is implemented using finite element analysis (FEA) with the general-purpose FEA software, ABAQUS. Specifically, the above-mentioned stiffness degradation law, damage initiation, and damage evolution are implemented using a VUMAT subroutine while the delamination damage is modeled by defining cohesive surface contacts between adjacent laminate plies. Note that finite element methods have been widely adopted for analyses of mechanics of composite laminates [56,57].

### 4.1. Model Verification Using a Benchmark Low Velocity Impact Problem

To verify the model, the low velocity impact problem reported by Ref. [52] is used as a benchmark. The reason for choosing this problem is due to the completeness of the experimental test results, including the load vs. time curve, the load vs. displacement curve, and the delamination patterns per layer. This problem has been widely used as a benchmark in many studies for the verification of low velocity impact models [10,49,53,58]. Below is a brief description of this benchmark problem, followed by the verification results.

**Figure 3:**
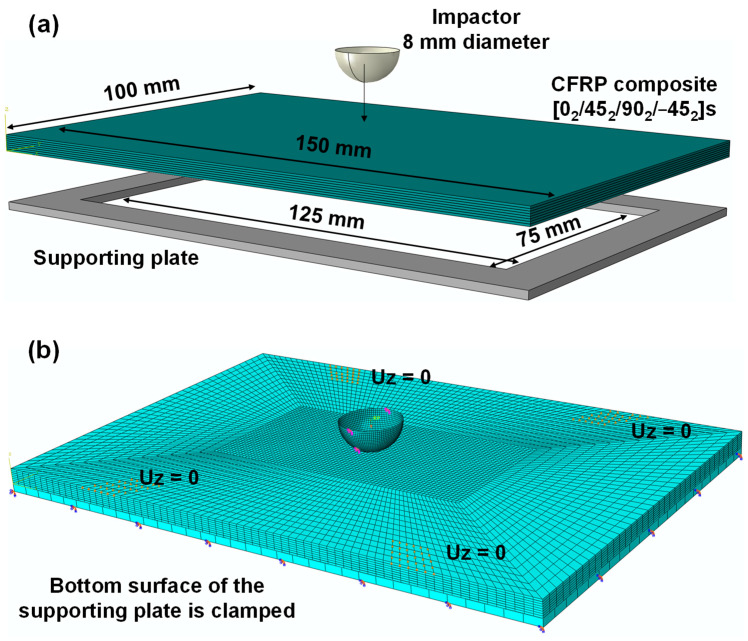
Verification of the low velocity impact model using a benchmark simulation problem for a CFRP composite laminate under an impact energy of 25 J: (**a**) problem setup and (**b**) mesh and boundary conditions used in the finite element analysis. Note: the x direction represents the longitudinal direction or direction 1, y direction represents the transverse direction or direction 2, and z direction represents the through-thickness direction or direction 3 in the laminate coordinate system.

The CFRP composite laminate considered in this benchmark is a T700CG/M21 carbon fiber epoxy resin composite laminate with a layup of [0_2_/45_2_/90_2_/−45_2_]_S_ and a dimension of 150 × 100 × 4.16 mm. The impactor is made of steel, has a mass of 2 kg, and has a semi-spherical head with a diameter of 16 mm. The velocity of the impactor is 5 m/s, representing an impact energy of 25 J. The composite laminate is placed on top of a supporting plate with an inner open-cut window of 125 × 75 mm. The impactor and the supporting plate are modeled as discrete rigid bodies using R3D4 elements while the CFRP composite laminate is modeled using the C3D8R elements (i.e., eight-node linear brick, reduced integration elements). Figure 3 shows the schematic of the problem setup and the mesh used for the impactor, the CFRP composite, and the supporting plate. The global seed sizes of the impactor and the supporting plate defined in ABAQUS are 0.5 and 3 mm, respectively. For the CFRP composite, the mesh size at the center region (72 × 36 mm) directly under the impactor is refined to 0.9 × 0.9 mm, whereas the mesh at the regions far away from the impact site is created using a global seed size of 3.5 mm to reduce the computational time. To model the delamination, the interfaces between each adjacent ply pairs are assigned using cohesive surface contacts. The material parameters, including the density, Young’s modulus, Poisson’s ratios, strength, fracture energy, as well as the interface properties, are taken from Ref. [49]. The verification results are discussed in the section below.

### 4.2. Model Verification Results

**Figure 4:**
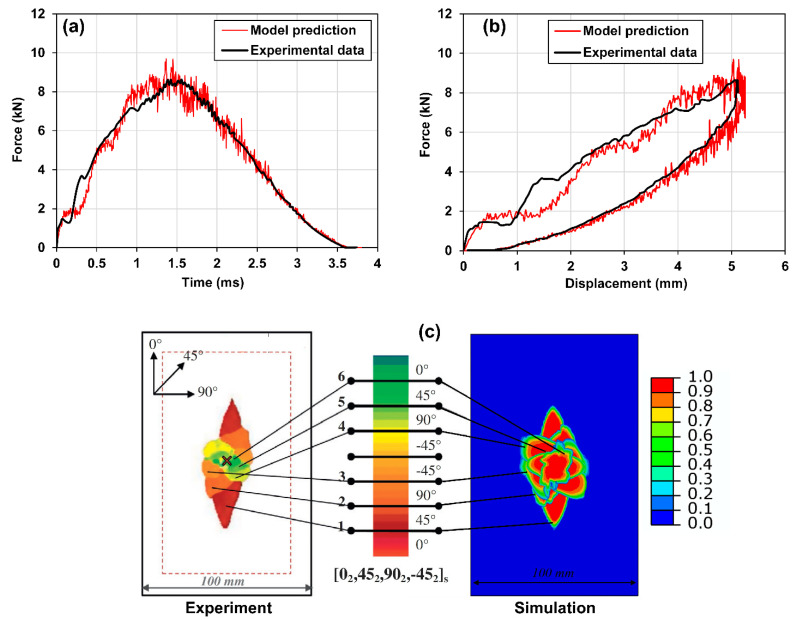
Verification of the low velocity impact model: comparison between the experimental and simulation results for a 25 J impact: (**a**) force history, (**b**) force vs. displacement, and (**c**) overlapped delamination areas in interfaces of the CFRP laminate between plies with different angles, where the experimental data were obtained using ultrasonic C-scan taken from Ref. [52]. The color legend of the C-scan contour (left) indicates the location of the delamination in the through-thickness direction, where greenish indicates the location near the top surface (impact side) while the reddish indicates the location near the bottom surface. The color legend in the simulation contour (right) indicates the degree of the predicted delamination, where the red color indicates complete delamination while the blue color indicates no delamination.

**Figure 5:**
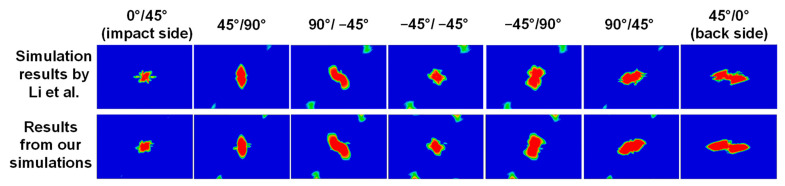
Verification of the low velocity impact model: comparison between simulation results reported in Ref. [49] (first row) and our simulation results (second row) for the same benchmark problem (i.e., low velocity impact at an impact energy of 25 J for a CFRP composite laminate of 150 × 100 mm and a layup of [0_2_/45_2_/90_2_/−45_2_]_S_). The contour plots from left to right show the delamination patterns at each interface of the composite laminate. The red color indicates complete delamination while the blue color indicates no delamination.

The model verification results are shown in Figure 4 and Figure 5. Specifically, Figure 4a shows the comparison between the simulation result of the force history during the impact and the experimental test result provided by Ref. [52]. As one can see, the predicted force history, including the peak load, the time to peak load, and the duration of the impact agree well with the experimental test data. Additionally, Figure 4b,c show comparisons of the force vs. displacement and the overlapped delamination patterns between the simulation results and the reported experimental test results, in which good agreements can be observed. Specifically, the experimental data of the delamination patterns are shown on the left side of Figure 4c. The data are taken from Ref. [52], which were obtained using an ultrasonic C-scan. The delamination patterns in interfaces of the composite laminate between plies with different angles are overlapped and illustrated in the C-scan contour. The color legend of the contour indicates the location of the delamination in the through-thickness direction, where the greenish color indicates a location near the top surface (i.e., impact side), while the reddish color indicates a location near the bottom surface. On the other hand, the predicted delamination patterns are shown on the right-hand side of Figure 4c, in which the color legend indicates the degree of delamination, where the red color indicates complete delamination while the blue color indicates no delamination.

To further illustrate the predictive accuracy of the low velocity impact model, the predicted ply-by-ply delamination patterns are compared with the simulation results reported by Ref. [52], as shown in Figure 5. It can be seen that the predicted delamination patterns are identical.

## 5. Results and Discussion

After the impact model was verified, it was employed to study the effect of laminate-level Poisson’s ratios, i.e., the combined effect of the **A**, **B**, and **D** matrices (see Equations (1) and (2)), on the low velocity impact behavior of the auxetic and non-auxetic IM7/977-3 layered CFRP composites. To achieve this, the same computational setup shown in Figure 3 was used, except that the number of layups was reduced from 16 layers to 10 layers, and the layups of the laminates follow those identified in Table 2. The ply-level engineering constants of the composite lamina used in the simulation studies are shown in Table 1. The simulations were conducted at three elevated impact energy levels, i.e., 3, 5, and 8 J. The choice of these three energy levels is because they were found, from preliminary trial-and-error simulation studies, to produce minimum, intermediate, and maximum damage without causing the laminates to penetrate.

### 5.1. Effect on the Global Response during Low Velocity Impact

**Figure 6:**
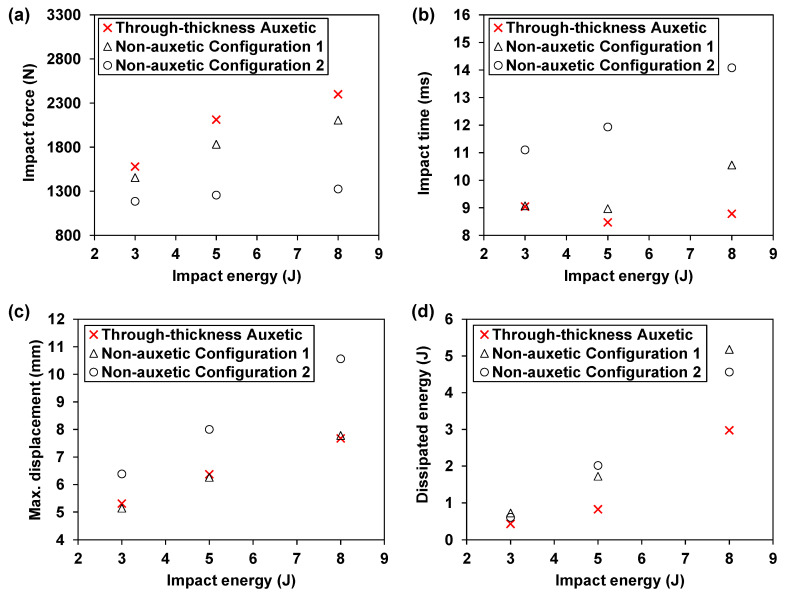
Effect of the through-thickness negative Poisson’s ratio on the global impact response: comparison between the auxetic and corresponding non-auxetic CFRP composite laminates: (**a**) impact load, (**b**) impact time, (**c**) maximum displacement, and (**d**) dissipated energy.

As shown in Figure 6a, the auxetic laminate shows consistently higher impact forces at all three energy levels, when compared to the non-auxetic counterparts. Meanwhile, the difference in the impact force between the auxetic laminate and the non-auxetic counterparts becomes more significant as the impact energy increases. Specifically, at 3 J, the impact force of the auxetic laminate is 8.7% and 33.3% higher than those of the non-auxetic Configuration 1 and Configuration 2 laminates, respectively. These differences increase to 14.0% and 81.0% as the impact energy increases from 3 J to 8 J. Moreover, it can be observed in Figure 6a that the predicted impact forces of the non-auxetic Configuration 1 laminate are much higher than those of non-auxetic Configuration 2 laminate and are relatively closer to those of the auxetic laminate. This is due to the higher effective moduli in the transverse and through-thickness directions of the non-auxetic Configuration 1 laminate than those of the Configuration 2 laminate (see Table 2). Note that mechanical impact is a contact problem where the effective contact modulus is a function of the through-thickness modulus [36]. Moreover, impact also involves biaxial bending where transverse stiffness also plays an important role. Therefore, the higher effective moduli in the transverse and through-thickness directions can cause a higher impact force.

In addition, the auxetic laminate shows consistently shorter impact times as shown in Figure 6b. Similar to the result of the impact force, the difference in the impact time between the auxetic laminate and the non-auxetic counterparts becomes more significant as the impact energy increases. Furthermore, the impact times of the non-auxetic Configuration 1 laminate are consistently much shorter than those of the non-auxetic Configuration 2 laminate and are closer to those of the auxetic laminate. This, together with the result of the impact force, implies that, although the through-thickness negative Poisson’s ratio increases the impact force and decreases the impact time, such an effect can be mitigated if the transverse or through-thickness effective moduli of the laminate decrease after the laminate layup is tuned to produce the through-thickness negative Poisson’s ratio.

Furthermore, the auxetic laminate shows consistently much lower maximum displacements than those of the non-auxetic Configuration 2 laminate, as depicted in Figure 6c. The reductions are 16.8%, 20.3%, and 27.4% at 3, 5, and 8 J, respectively. This indicates that producing the through-thickness negative Poisson’s ratio is beneficial for reducing the maximum displacement during low velocity impact. Moreover, this positive effect is more significant as the impact energy increases. For the Configuration 1 laminate, the maximum displacements are slightly lower (i.e., 3.2% and 1.7%) than those of the auxetic laminate at 3 and 5 J. However, when the impact energy rises to 8 J, the maximum displacement of the Configuration 1 non-auxetic laminate exceeds that of the auxetic laminate by 1.4%. The result suggests that both the negative Poisson’s ratio and the increased effective modulus (i.e., the transverse modulus of Configuration 1 laminate is higher than that of the auxetic laminate, see Table 2) are beneficial for reducing the maximum displacement. Specifically, at lower energy levels (i.e., 3 and 5 J), the increased transverse effective modulus is a more dominating factor, whereas at a higher energy level (i.e., 8 J), the effect of the through-thickness negative Poisson’s ratio becomes more significant than the effect of the increased modulus, which contributes to a more significant reduction of the maximum displacement.

Moreover, Figure 6d illustrates that the auxetic laminate consistently exhibits lower dissipated energies than those of the non-auxetic laminates. Since the energy dissipation is closely related to the damage behavior of the composite laminates, the effect of the through-thickness negative Poisson’s ratio on the damage behaviors, including delamination and fiber and matrix damage, is examined in the following sections.

### 5.2. Effect on the Delamination Damage

**Figure 7:**
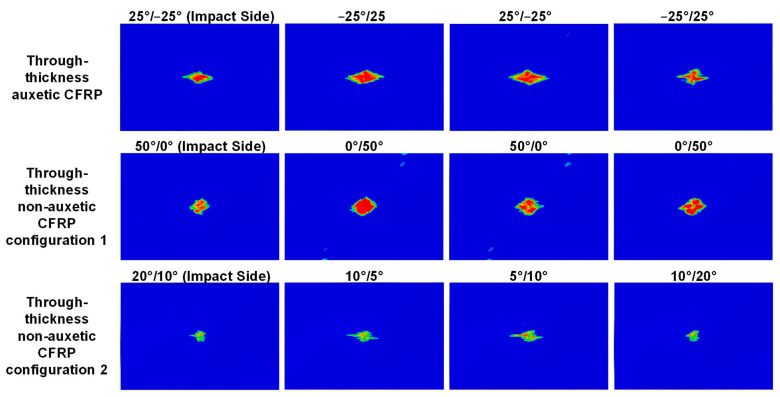
Effect of the through-thickness negative Poisson’s ratio on the delamination: comparison of predicted delamination pattern in each interface of auxetic CFRP composite (layup: [25_2_/−25_2_/25_2_/−25_2_/25_2_], results shown in the first row) and the corresponding non-auxetic laminates in two configurations (layups: [50_2_/0_2_/50_2_/0_2_/50_2_] and [20_2_/10_2_/5_2_/10_2_/20_2_], results shown in the second and third rows), at an 8 J impact. The red color indicates complete delamination while the blue color indicates no delamination.

The through-thickness negative Poisson’s ratio, $\nu_{13}^{e}$, have restrained the delamination growth in the transverse direction but encouraged the growth in the longitudinal direction. Figure 7 illustrates the comparison of the predicted delamination patterns in each interface of the auxetic laminate and the corresponding non-auxetic laminates at an impact energy of 8 J. Specifically, the delamination patterns in the auxetic laminate follow a diamond shape where the transverse diagonal length is much shorter than the longitudinal diagonal length. The delamination patterns in the non-auxetic Configuration 1 laminate also exhibit a diamond shape, but the lengths of the two diagonals are almost identical. As for the non-auxetic Configuration 2 laminate, the shapes of delamination at the top and bottom interfaces are like those of the non-auxetic Configuration 1 laminate, while the shapes at the two middle interfaces are similar to those of the auxetic laminate. 

**Figure 8:**
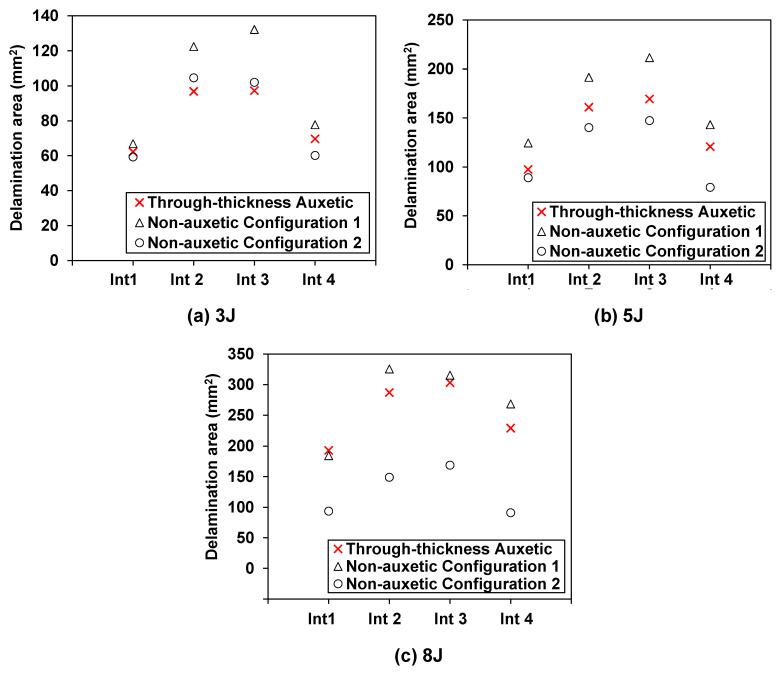
Effect of the through-thickness negative Poisson’s ratio on the delamination: comparison of the predicted delamination area in each interface of the auxetic and corresponding non-auxetic CFRP composite laminates at: (**a**) 3 J, (**b**) 5 J, and (**c**) 8 J, where “int” in the horizontal axis denotes the interface of the composite laminate.

Figure 8 provides a quantitative comparison of the delamination area between the auxetic and non-auxetic laminates. At an impact energy of 3 J, the delamination areas of the auxetic laminate at all interfaces are close to those of the non-auxetic Configuration 2 laminate, as shown in Figure 8a. At 5 and 8 J, the auxetic laminate exhibits larger delamination areas than the non-auxetic Configuration 2 laminate, as shown in Figure 8b,c. The difference increases as the impact energy increases. At 8 J, the delamination areas of the auxetic laminate are 2.1, 1.9, 1.8, and 2.5 times those of the non-auxetic Configuration 2 laminate at the four interfaces, respectively. Results indicate that producing the through-thickness negative Poisson’s ratio does not present a clear advantage in mitigating the delamination damage.

As for the non-auxetic Configuration 1 laminate, the delamination areas are much larger than those of the non-auxetic Configuration 2 laminate and are generally larger than those of the auxetic laminate, as shown in Figure 8a–c. This is due to the high transverse effective modulus of the non-auxetic Configuration 1 laminate. Additionally, it can be observed that, as the impact energy increases, the difference in the delamination areas between the non-auxetic Configuration 1 laminate and the auxetic laminate becomes smaller and smaller. At 8 J, the delamination areas of the two laminates are identical, as shown in Figure 7 and Figure 8c. This implies that both producing through-thickness negative Poisson’s ratio and increasing the transverse effective modulus could lead to an adverse effect on the delamination propagation. As the impact energy increases, the adverse effect due to the through-thickness negative Poisson’s ratio becomes more pronounced. 

Note that although the delamination damage becomes more pronounced as the impact energy increases, the dissipated energy, as shown in Figure 6d, is still consistently lower for the auxetic laminate. This indicates that the other damage modes (e.g., fiber and matrix damage) in the auxetic laminate are more confined as the impact energy increases, when compared to the non-auxetic laminates, which will be discussed as follows.

### 5.3. Effect on the Matrix and Fiber Damage

**Figure 9:**
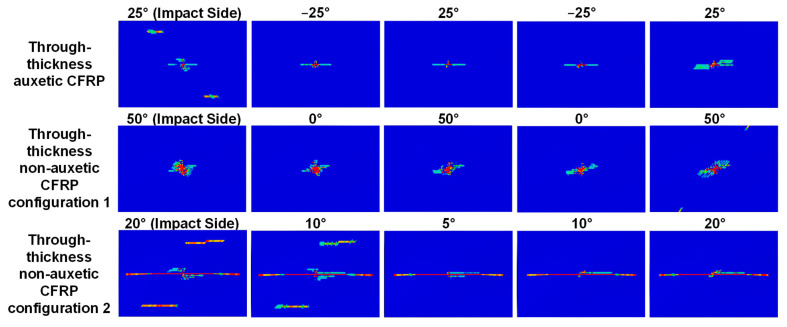
Effect of the through-thickness negative Poisson’s ratio on the matrix tensile damage: comparison of predicted matrix tensile damage pattern in each ply of the auxetic CFRP composite (layup: [25_2_/−25_2_/25_2_/−25_2_/25_2_], results shown in the first row) and the corresponding non-auxetic laminates in two configurations (layups: [50_2_/0_2_/50_2_/0_2_/50_2_] and [20_2_/10_2_/5_2_/10_2_/20_2_], results shown in the second and third rows), at an 8 J impact. The red color indicates complete damage while the blue color indicates no damage.

**Figure 10:**
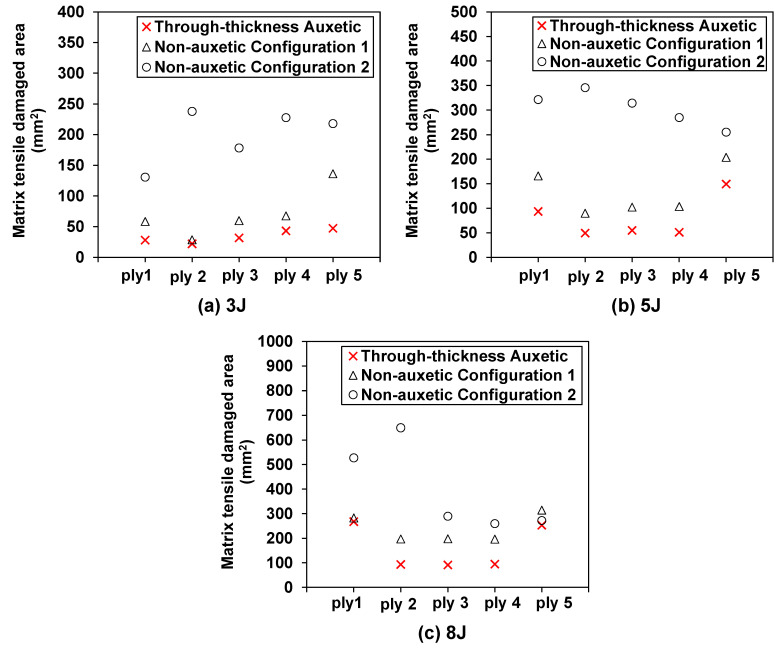
Effect of the through-thickness negative Poisson’s ratio on the matrix tensile damage: comparison of the predicted damage area in each ply of the auxetic and non-auxetic CFRP composite laminates at: (**a**) 3 J, (**b**) 5 J, and (**c**) 8 J.

The matrix tensile, the fiber tensile, and the matrix compressive damage are commonly observed damage modes in CFRP composites under low velocity impact (fiber compressive damage is often negligible [49]). The effect of the through-thickness negative Poisson’s ratio on the matrix tensile damage is illustrated in Figure 9 and Figure 10. Specifically, Figure 9 shows the comparison of the predicted patterns of the matrix tensile damage in each ply of the auxetic laminate and non-auxetic laminates at an impact energy of 8 J. It can be clearly observed that the auxetic laminate exhibits consistently much smaller matrix tensile damage in all plies, when compared to the two non-auxetic laminates. This is also true for cases with impact energies of 3 and 5 J, as shown in Figure 10. 

Figure 10a–c show that the auxetic laminate exhibits consistently much smaller matrix tensile damage areas in all plies, when compared to the non-auxetic laminates at all impact energy levels. For instance, at 8 J, the predicted matrix tensile damage areas in the auxetic laminate are shown to reduce by 49.3%, 86.5%, 68.5%, 63.6%, and 7.0%, in the five plies, respectively, in comparison to the non-auxetic Configuration 1 laminate, and are shown to reduce by 5.2%, 5.3%, 54.0%, 51.8%, and 20.0%, in the five plies, respectively, in comparison to the non-auxetic Configuration 2 laminate. This suggests that producing the through-thickness negative Poisson’s ratio is beneficial in suppressing the propagation of the matrix tensile damage, as the contraction in the through-thickness direction during the impact leads to the contraction in the in-plane direction caused by the negative Poisson’s ratio (see Figure 1), thereby mitigating the tensile damage. Furthermore, although the non-auxetic Configuration 1 laminate has a higher transverse effective modulus than the auxetic laminate, the predicted matrix tensile damage areas are still consistently much larger than those of the auxetic laminate. This implies that the matrix tensile damage is much more sensitive to the negative Poisson’s ratio than the increase of the transverse effective modulus.

**Figure 11:**
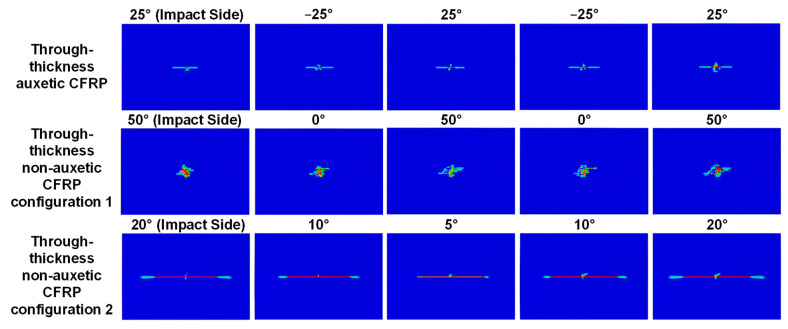
Effect of the through-thickness negative Poisson’s ratio on the fiber tensile damage: comparison of the predicted fiber tensile damage pattern in each ply of the auxetic CFRP composite laminate (layup: [25_2_/−25_2_/25_2_/−25_2_/25_2_], results shown in the first row) and the corresponding non-auxetic CFRP composite laminates (layups: [50_2_/0_2_/50_2_/0_2_/50_2_] and [20_2_/10_2_/5_2_/10_2_/20_2_], results shown in the second and third rows), at an 8 J impact. The red color indicates complete damage while the blue color indicates no damage.

**Figure 12:**
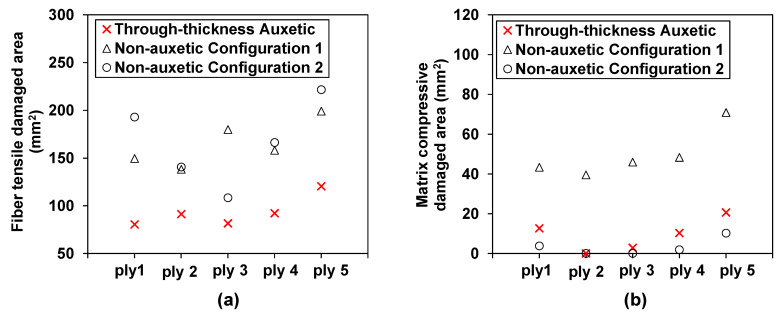
Effect of the through-thickness negative Poisson’s ratio on the fiber tensile damage and matrix compressive damage: comparison of the predicted damage areas in each ply of the auxetic laminate and the corresponding non-auxetic CFRP composite laminates at an impact energy of 8 J: (**a**) fiber tensile damaged area and (**b**) matrix compressive damaged area.

The influence of the through-thickness negative Poisson’s ratio on the fiber tensile damage is shown in Figure 11 and Figure 12a. As illustrated in Figure 11, the auxetic laminate shows consistently much reduced fiber tensile damage than that of the two non-auxetic laminates. Note that the fiber tensile damage is negligible at lower impact energies (i.e., 3 and 5 J) and therefore only the predictions at 8 J are presented. It can be seen from Figure 12a, the reductions in the fiber tensile damaged areas of the auxetic laminate are 46.2%, 34.0%, 54.7%, 41.7%, and 39.5% for the five plies, respectively, in comparison to those of the non-auxetic Configuration 1 laminate, and are 58.4%, 35.1%, 24.8%, 44.6%, and 45.7% for the five plies, respectively, in comparison to those of the non-auxetic Configuration 2 laminate. The results suggest that producing the through-thickness negative Poisson’s ratio significantly reduces the fiber tensile damage. This could also be due to the contraction in the in-plane direction caused by the negative Poisson’s ratio during the impact event (see Figure 1).

The auxetic laminate generally shows a larger matrix compressive damaged area than the non-auxetic Configuration 2 laminate, as shown in Figure 12b. Specifically, at ply 1, ply 3, and ply 5, the matrix compressive damaged areas of the auxetic laminate are 3.4, 5.3, and 2.0 times of those of the non-auxetic Configuration 2 laminate (note that ply 2 and ply 3 have no matrix compressive damage in the non-auxetic Configuration 2 laminate). This implies that the through-thickness negative Poisson’s ratio could unfavorably extend the matrix compressive damaged areas. This could be because the contraction in the in-plane direction during the impact event caused by the negative Poisson’s ratio exacerbates the compressive damage (see Figure 1). As for the non-auxetic Configuration 1 laminate, interestingly, the matrix compressive damaged areas are significantly larger than those of the auxetic laminate and those of the non-auxetic Configuration 2 laminate. This implies that the matrix compressive damaged area is sensitive to the transverse effective modulus. As it increases, the laminate is prone to experience more extensive matrix compressive damaged areas.

To briefly summarize, the through-thickness negative Poisson’s ratio shows large influences on the global impact response and damage behavior due to the unique triaxial state of stresses produced in the auxetic laminate. For the global response, the simulation results suggest that producing the negative Poisson’s ratio in the laminate could result in an increase of the impact force and reductions in the impact time, maximum displacement, and the dissipated energy. For the damage behavior, the negative Poisson’s ratio could lead to increases in the delamination areas and matrix compressive damaged areas and significant reductions in the matrix tensile and fiber tensile damaged areas. 

## 6. Conclusions

The effect of laminate-level through-thickness negative Poisson’s ratio on the low velocity impact behavior of the CFRP composite laminate is investigated using numerical simulations. The layups of the auxetic laminates are identified based on the Classical Lamination Theory. The auxetic laminate has a laminate-level through-thickness Poisson’s ratio of −0.327. The layups of the non-auxetic counterpart laminates are determined by matching the effective moduli in three directions while allowing them to produce positive Poisson’s ratios. The main conclusions are summarized below.

The through-thickness negative Poisson’s ratio reflects the combined effect of the **A**, **B**, and **D** stiffness matrices. It largely influences the global impact response and the damage behavior. The auxetic laminate with a through-thickness negative Poisson’s ratio shows consistently higher impact forces, shorter impact times, reduced maximum displacements, and lower dissipated energies at elevated impact energy levels. For the damage behavior, the through-thickness negative Poisson’s ratio does not present a clear advantage in mitigating the delamination damage, but it results in consistent and significant reductions in the matrix tensile damage and fiber tensile damage (i.e., 40% reduction in matrix tensile damage on average and 42% reduction in fiber tensile damage on average). At the same time, it could unfavorably yield an increase in the matrix compressive damage (i.e., 1.5 times increase). 

Future studies are recommended to investigate the effect of the elevated auxeticity on the impact behavior and if the findings presented in this study are still valid for thick auxetic laminates, as well as the interlaminar cohesive behaviors of auxetic laminates.

## Figures and Tables

**Table 1:** Material properties of IM7/977-3 CFRP composites [47,48,49].

**Composite lamina properties**	Density	*ρ* = 1600 kg/m^3^
	Elastic moduli	*E*_11_ = 159 GPa, *E*_22_ = *E*_33_ = 9.2 GPa
		*G*_12_ = *G*_13_ = 4.37 GPa, *G*_23_ = 2.57 GPa
	Poisson’s ratio	*ν*_12_ = *ν*_13_ = 0.253, *ν*_23_ = 0.456
	Strength	*X_T_* = 2275 MPa, *X_C_* = 1680 MPa, *Y_T_* = 64 MPa, *Y_C_* = 168 MPa. *S_xy_* = 121 MPa, *S_yz_* = *S_zx_* = 127 MPa
	Fracture energy	*G_ft_* = 133 N/mm, *G_fc_ =* 40 N/mm, *G_mt_ =* 0.6 N/mm, *G_mc_ =* 2.1 N/mm
**Interface properties**	Modulus	*E* = 5 GPa
	Strength	*N* = *S* = 30 MPa
	Fracture energy	$G_{n}^{C}$ = 0.6 N/mm (normal), $G_{s}^{C}$ = 2.1 N/mm (shear)

**Table 2:** Layups of the auxetic laminate and the corresponding non-auxetic counterpart composite laminates in two configurations.

	Through-Thickness Auxetic CFRP Laminate	Configuration 1: Non-Auxetic Counterpart CFRP Laminate (with Strictly Matched $E_{1}^{eff}$ and $E_{3}^{eff}$)	Configuration 2: Non-Auxetic Counterpart CFRP Laminate (with Weakly Matched $E_{1}^{eff}$, $E_{2}^{eff}$, and $E_{3}^{eff}$)
Layup	[25_2_/−25_2_/25_2_/−25_2_/25_2_]	[50_2_/0_2_/50_2_/0_2_/50_2_]	[20_2_/10_2_/5_2_/10_2_/20_2_]
$\nu_{13}^{e}$	−0.327	0.264	0.260
$E_{1}^{eff}$ (GPa)	70.83	70.33 (−0.7%)	71.88 (+1.5%)
$E_{2}^{eff}$ (GPa)	9.45	14.23 (+50.6%)	9.46 (+0.1%)
$E_{3}^{eff}$ (GPa)	9.95	9.95 (0)	9.24 (−7.1%)

## Data Availability

The VUMAT subroutine and datasets generated during and/or analyzed during the current study are available from the corresponding author on reasonable request.
